# Phase-engineering compact and flexible CsPbBr_3_ microcrystal films for robust X-ray detection†

**DOI:** 10.1039/d3tc01903a

**Published:** 2023-12-11

**Authors:** Lotte Clinckemalie, Bapi Pradhan, Roel Vanden Brande, Heng Zhang, Jonathan Vandenwijngaerden, Rafikul Ali Saha, Giacomo Romolini, Li Sun, Dirk Vandenbroucke, Mischa Bonn, Hai I. Wang, Elke Debroye

**Affiliations:** a Department of Chemistry, KU Leuven Celestijnenlaan 200F 3001 Leuven Belgium elke.debroye@kuleuven.be bapi.pradhan@kuleuven.be; b Max Planck Institute for Polymer Research 55128 Mainz Germany; c cMACS, Department of Microbial and Molecular Systems, KU Leuven Celestijnenlaan 200F 3001 Leuven Belgium; d Agfa-Gevaert Septestraat 27 B-2640 Mortsel Belgium

## Abstract

All-inorganic CsPbBr_3_ perovskites have gained significant attention due to their potential in direct X-ray detection. The fabrication of stable, pinhole-free thick films remains challenging, hindering their integration in durable, large-area high-resolution devices. In this study, we propose a facile strategy using a non-conductive polymer to create a flexible, compact thick film under ambient conditions. Furthermore, we investigate the effect of introducing the 2D CsPb_2_Br_5_ phase into CsPbBr_3_ perovskite crystals on their photophysical properties and charge transport. Upon X-ray exposure, the devices consisting of the dual phase exhibit improved stability and more effective operation at higher voltages. Rietveld refinement shows that, due to the presence of the second phase, local distortions and Pb-vacancies are introduced within the CsPbBr_3_ lattice. This in turn presumably increases the ion migration energy barrier, resulting in a very low dark current and hence, enhanced stability. This feature might benefit local charge extraction and, ultimately, the X-ray image resolution. These findings also suggest that introducing a second phase in the perovskite structure can be advantageous for efficient photon-to-charge carrier conversion, as applied in medical imaging.

## Abstract 2

In this article, the introduction of a second phase with controlled defect states in the perovskite structure is shown to be advantageous for stable, high-energy X-ray detection.

## Graphical abstract

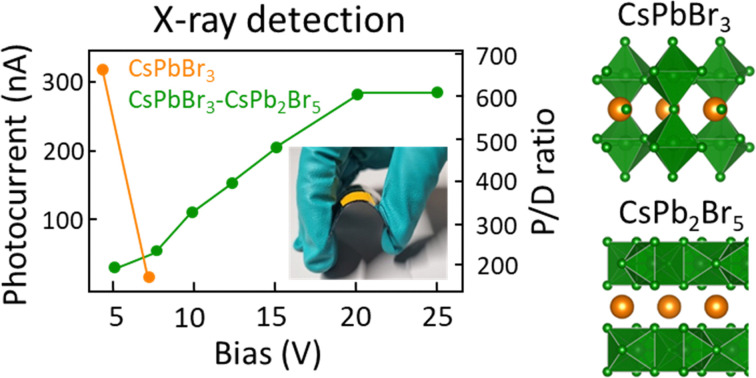

## Introduction

Metal halide perovskite semiconductor materials have attracted significant attention owing to their exceptional optoelectronic properties, such as strong light absorption,^1^ high carrier mobility,^2^ and long electron–hole diffusion lengths.^3^ Their low-cost fabrication, in combination with the ability to tune their optical and electronic properties through their compositional and morphological flexibility, have made diverse perovskite compounds the focus of intense study for different applications such as solar cells,^4^ light emission applications (LEDs)^5,6^ and photo- and X-ray detection.^7,8^ X-ray detection is a crucial technology in many areas, such as medical imaging, industry and security screening, and material analysis.^9,10^ Over the years, the soft X-ray industry is transitioning from indirect detectors, in which a scintillator material is coupled to a photodetector, to more sensitive direct-conversion X-ray detectors.^11^ Most commercially available detectors are based on a semiconductor material, such as cadmium (zinc) telluride, or amorphous selenium (a-Se), which is currently used in mammography. However, the high cost and complex fabrication processes, or operational instability of these materials in combination with the low X-ray attenuation of a-Se constitute a significant bottleneck for their commercial usage.^12–16^

All-inorganic CsPbBr_3_ perovskite has demonstrated significant potential in this field due to its large X-ray attenuation coefficient arising from the high average atomic number combined with intrinsic defect tolerance and good charge collection efficiency.^15,17–20^ It exhibits an intermediate bandgap energy, which is advantageous compared to the high bandgap of CsPbCl_3_. Additionally, it has an improved room temperature stability compared to CsPbI_3_, which only exhibits its photoactive perovskite phase above 350 °C.^21–23^ Despite its many advantages, several challenges need to be addressed before it can be fully commercialized for X-ray detection applications. One of the main obstacles is the stability of the material, as it is sensitive to moisture and heat, and prone to degradation, promoting the formation of detrimental defects and sacrificing the device performance.^24–27^ Furthermore, high-quality, phase-pure CsPbBr_3_ crystals are often synthesized *via* complex, prolonged and high-temperature synthesis methods, making it difficult to produce them on a large scale.^28–30^ Finally, fabricating high-quality, thick perovskite films remains a great challenge, primarily due to the presence of numerous pinholes that often occur as a result of solvent evaporation in the typical solution-based processes.^19^

In this paper, we developed a facile, room-temperature microcrystal synthesis strategy without the need of ligands and with precise control over crystal purity. Furthermore, a compact, thick film was fabricated in ambient conditions, using a polymer binder as a stabilizer. We show that control over the phase purity is a promising route to improve the stability of CsPbBr_3_. More specifically, we investigated how introducing a secondary phase, *e.g.* two-dimensional (2D) CsPb_2_Br_5_, positively affects the structural and photophysical properties. Due to the presence of ∼23% of the CsPb_2_Br_5_ phase, Pb defects are formed in the dual-phase material, which inturn enhances crystal symmetry and material stability. While this effect somewhat reduces the charge carrier mobility, it results in a much-enhanced charge carrier lifetime. These properties lead to improved stability and operation at higher voltage regimes, which is expected to benefit charge extraction and can ultimately improve the X-ray image resolution. These findings suggest that introducing shallow defect levels in the perovskite lattice structure can benefit energy conversion applications.

## Results and discussion

**Fig. 1:**
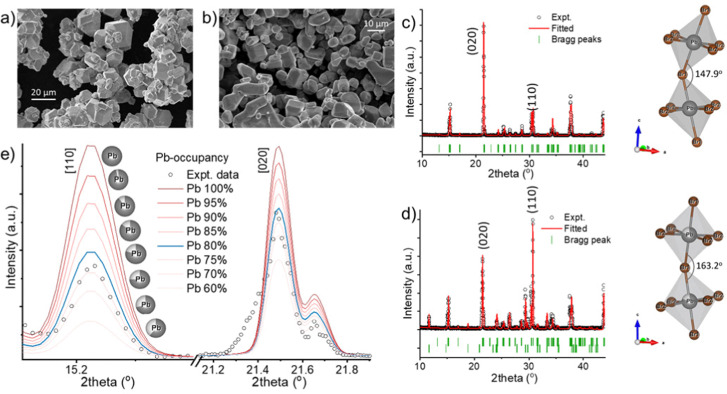
Scanning electron microscope (SEM) image of (a) phase pure CsPbBr_3_ and (b) dual-phase CsPbBr–CsPb_2_Br_5_ microcrystals (MCs). Rietveld refined XRD data of (c) pure CsPbBr_3_ and (d) the dual-phase compound showcasing the experimental data (black circles), fitted data (red line), and Bragg peaks (green scattered lines). The octahedral bond angles between Pb–Br1–Pb are shown in the right panels, respectively. (e) 〈110〉 and 〈020〉 planes of the CsPbBr_3_ phase in the dual-phase compound. The black circles depict the experimental data, while the curves represent the fitted data. Different curves signify the fitting of varying Pb occupancies in CsPbBr_3_. In the inset, the black part of the spherical balls represents the Pb occupancy, and the white area indicates Pb vacancies.

To synthesize gram amounts of CsPbBr_3_ microcrystals (MCs), a stoichiometric molar ratio of CsBr (5 mmol) and PbBr_2_ (5 mmol) is first separately dissolved in 6 mL and 4 mL of 48 w% HBr, respectively, by sonication. These solutions are then added simultaneously and dropwise over 10 minutes to 1.5 mL of 48 w% HBr under ambient conditions and constant stirring. Finally, the crystals are centrifuged (5 min, 3500 rpm), washed with ethanol and vacuum dried at 60 °C. This results in icosahedral-shaped microcrystals with an average size of ∼15 μm (Fig. 1(a)). Upon slightly changing the synthesis procedure, we discovered the formation of a secondary phase after adding demi H_2_O in a 1 : 4 volume ratio to the precursor mixture (for details, see ESI†). This results in microcrystals with a slightly rounded shape and an average size of ∼10 μm (Fig. 1(b)). X-ray diffraction (XRD) confirms the formation of phase-pure orthorhombic CsPbBr_3_ crystals (Fig. 1(c)).^28^ When a small amount of water is introduced with in the synthesis protocol then the XRD pattern of resultant MCs identifies the appearances of 2D tetragonal CsPb_2_Br_5_ secondary crystal phases (Fig. 1(d) and Fig. S2, S3, ESI†).^28,31^ Inside the polymer matrix, the crystal structure and relative peak intensities do not change substantially (Fig. S2, ESI†), implying that the solution-processed thick film preserves the crystal structure and phase mixing. Rietveld refinement of the diffraction patterns has been performed by using FULLPROF suite software. The phase pure sample was found to reveal a strong preference for the 〈020〉 plane orientation. Furthermore a Pb–Br1–Pb bond angle of 147.9° and a Pb–Br2–Pb bond angle of 157.6° has been determined (Fig. 1(c) and Fig. S4, ESI†). For the dual-phase sample, it is found to comprise 23% CsPb_2_Br_5_ and 77% CsPbBr_3_. Upon the addition of water, the CsPbBr_3_ MCs show a favored growth in the direction along the 〈110〉 plane at 2*θ* = 30.7°, when compared to the 〈020〉 plane peak (2*θ* = 21.5°) in the all-acid synthesis, indicating the presence of lead vacancies.^32,33^ The appearance of the secondary phase induces around 20% Pb vacancies within the CsPbBr_3_ phase, confirmed *via* Rietveld refinement. Fig. 1(e) displays fitted data aligning well with an 80% Pb occupancy, affirming 20% of Pb vacancies. These vacancies induce local lattice distortions, consequently raising the ion migration energy barrier.^34^ For the dual-phase sample, the bond angle of Pb–Br1–Pb and Pb–Br2–Pb was determined to be 163.2° and 156.2°, respectively (Fig. 1(d) and Fig. S4, ESI†). The octahedral tilting factor (*ψ*) is pivotal for perovskite stability, enhancing crystal symmetry and system stability. Calculated as *ψ* = (180 − *ϕ*)/2, reduced tilting (*ϕ*) in Pb–Br1–Pb bonds is demonstrated in both samples, while Pb–Br2–Pb bonds remain stable (Fig. S4, ESI†).^35^ This reduction enhances stabilization, especially in dual-phase compounds, indicating a shift towards higher symmetry. Consequently, the room temperature orthorhombic phase becomes more stable. Further careful comparison of the XRD patterns of pure and dual-phase samples shows that the first two prominent peaks do not exhibit a significant shift, but the third peak (highlighted in red) has slightly moved to a higher angle, signifying lattice distortion within the CsPbBr_3_ phase (Fig. S5, ESI†). Additionally, for the dual-phase compound, the first and second peak appear slightly broader, potentially due to lattice distortion induced by the presence of the CsPb_2_Br_5_ phase in the composite system. The calculated lattice constants of pure CsPbBr_3_ are *a* = 8.21 Å, *b* = 8.27 Å, *c* = 11.77 Å (space group *Pbnm*); and that of dual-phase CsPbBr_3_ are *a* = 8.20 Å, *b* = 8.26 Å, *c* = 11.76 Å (space group *Pbnm*) and CsPb_2_Br_5_ are *a* = *b* = 8.49 Å, *c* = 15.26 Å (space group *I*4/*mcm*), implying a minimal lattice mismatch at the interface due to the creation of the dislocation. The strain relaxation within these soft ionic solids with a small Young modulus presumably occurs through strongly coupled alternating Cs^+^ and (Pb_2_Br_5_)^−^ layers, as well as *via* atomic-level cation rearrangement.^36,37^

**Fig. 2:**
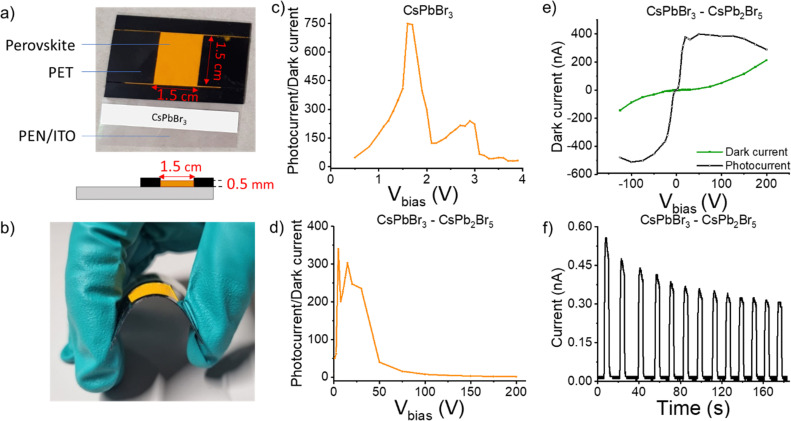
(a) Photograph of a compact thick film fabricated by the proposed coating method on a conductive substrate (top) and a schematic diagram of the cross-section of the perovskite microcrystal film (bottom). (b) Photograph of the compact thick film fabricated in the lab, showing its flexibility. Photo-to-dark current ratio upon X-ray exposure of (c) phase-pure CsPbBr_3_ and (d) dual-phase CsPbBr_3_–CsPb_2_Br_5_ films in function of the applied voltage. (e) Dark current (green) and photocurrent (black) of the dual-phase film. (f) Photo-switching measurements (10 cycles) on the dual-phase film at an applied voltage of 5 V.

Towards scalable deposition for large-area X-ray detection devices, we developed a new strategy to create flexible and uniform thick films consisting of perovskite MCs. In this strategy, a non-conductive polymer is used to guarantee a dense crystal packing to assure good contact between the MC particles while inhibiting the generation of cracks. Devices of both samples are fabricated to compare their response to X-ray exposure. The sample holders are fabricated by attaching polyethylene terephthalate (PET) sheets on a conductive polyethylene naphtalate (PEN) substrate, which is precoated with indium tin oxide (ITO) to create a ∼500 μm deep 1.5 × 1.5 cm^2^ square cavity (Fig. 2(a)). A 10 w% polyvinyl butyral (PVB) in isopropanol (IPA) is prepared as polymer binder solution. Next, a dispersion of 1 g of both the phase-pure and dual-phase perovskite microcrystals, 0.3 g of IPA and 0.3 g of the PVB polymer binder solution are mixed. Then, 0.3 g of this mixture is transferred into the square cavity so that it fully covers the entire surface. The films are then allowed to harden under ambient conditions due to the partial evaporation of the solvent. Finally, the remaining solvent is removed by drying the films in an oven at 60 °C for one hour, resulting in the desired flexible, compact films with a smooth surface allowing good contact for opto-electric measurements (Fig. 2(a), (b) and Fig. S6, ESI†).

As a proof-of-concept for X-ray detection, the devices are measured in PEN/ITO/perovskite/conductive silicone configuration. The distance between the X-ray source and the device is kept constant at 95 cm, mimicking a real-live X-ray imaging investigation. The generated current is probed in dark and while illuminated by an X-ray pulse (tungsten anode, 70 keV, 20 mA) under various bias voltages (*V*_bias_). The photo-to-dark current (P/D) ratio is then plotted as a function of *V*_bias_ to determine the voltage at which the photocharges are most efficiently collected. A high P/D ratio of 750 is calculated for the phase-pure MCs at a *V*_bias_ of 1.6 V (Fig. 2(c)). To assess the device performance, the detection sensitivity (*R*) is calculated by the following formula:
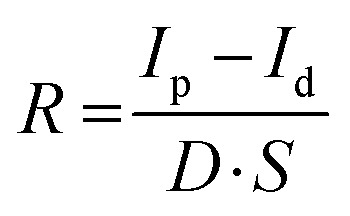
where *I*_p_ and *I*_d_ are the photocurrent and dark current, *D* is the X-ray dose rate and *S* is the effective area of the device (2.25 cm^2^).^38,39^ This results in a detection sensitivity of 22.05 μC Gy^−1^ cm^−2^ under a bias voltage of 4 V. The detection sensitivity is comparable to currently commercially available a-Se photodetectors.^12,40^ On the other hand, the device is found to be unstable upon further increasing bias and degrades rapidly during the experiment, as highlighted by the fast decrease in P/D ratio. Due to the high energy X-ray radiation and applied voltage, a plausible scenario is that ionic defects are readily formed, resulting in prominent ion migration and, thus, an increase in dark current.^41^ It has been demonstrated before that Br^−^ ions are released from the structure and migrate towards the positively biased electrode. This triggers degradation pathways and leads to a substantial decrease in charge collection.^42^ As the increase in dark current is so dramatic, no photo-switching measurements could be performed for the phase-pure sample.

For the device fabricated out of dual-phase MCs, a sensitivity of 1.25 μC Gy^−1^ cm^−2^ at 4 V is measured. Interestingly, upon increasing the voltage to 20 V, the P/D ratio reaches 250 (Fig. 2(d)), corresponding to a much-improved detection sensitivity of 15.42 μC Gy^−1^ cm^−2^. While this value is slightly lower compared to the phase-pure MCs, we show that the existence of the secondary phase, belonging to the 2D CsPb_2_Br_5_, substantially enhances the operational stability and tolerance of CsPbBr_3_ to higher voltage X-ray detection. Working towards implementation in high-end devices, operation at higher voltages is expected to benefit charge extraction, and improved device stability in these regimes is strongly required. Moreover, charge carriers can diffuse laterally and spread over multiple device pixels, leading to information blur. By applying a higher voltage, the lateral diffusion of the charge carriers can be limited, reducing cross-talk between different pixels, and ultimately improving the X-ray image resolution.^12,43,44^

Studying the dual-phase MCs into more detail, it can be observed that the photocurrent exhibits a steep curve until a *V*_bias_ of 50 V, while the dark current remains low over that same range (Fig. 2(e)). This means that even at high applied voltages, ion migration – which is the major cause of dark current – is substantially suppressed, which is generally a challenge for perovskite materials.^45,46^ It has been proposed that the lattice distortion formed due to the presence of the second phase, when compared to the phase-pure CsPbBr_3_ MCs, increases the ion migration energy barrier, resulting in a lower dark current.^47^ Photo-switching measurements are performed at an applied voltage of 5 V to test the repeatability of the device response (Fig. 2(f)). After 10 cycles, the generated photocurrent still equals 60% of the initially generated current. To confirm X-ray detection stability, a dose variation linearity test was conducted at a constant bias of 30 V. Increasing the X-ray dose from 0–70 μGy s^−1^ resulted in a linear rise in photocurrent, verifying the robustness of the dual-phase perovskite structure under these conditions (Fig. S7, ESI†). Furthermore, *in situ* synchrotron-based GIWAXS measurements demonstrated that the dual-phase film imparts high structural robustness over a constant X-ray exposure of 30 min (Fig. S8, ESI†). In the dual-phase CsPbBr_3_–CsPb_2_Br_5_ film, the primary phase remains consistent throughout the entire 30 min X-ray exposure. The phase-pure film undergoes peak splitting upon continued X-ray exposure, most probably attributed to partial beam damage (Fig. S8a, ESI†). Notably, this is a proof-of-concept device, and further optimization is needed to enhance the operation stability, which is beyond the scope of this work. However, this behavior confirms that the dual-phase CsPbBr_3_–CsPb_2_Br_5_ is a promising material composite for X-ray detection with long-term operational stability, particularly at high voltages.

**Fig. 3:**
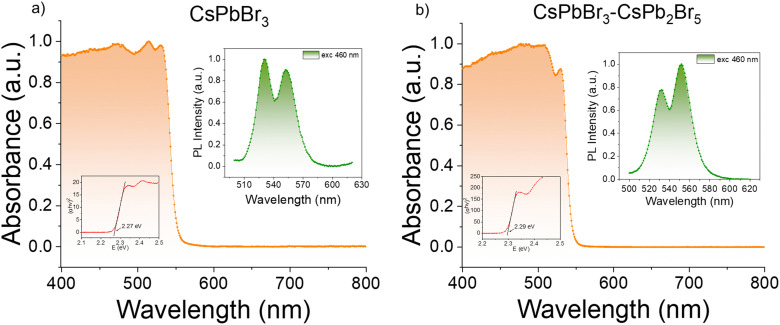
Absorption spectra of (a) phase-pure CsPbBr_3_ and (b) dual-phase CsPbBr_3_–CsPb_2_Br_5_. Tauc plots are shown in the lower insets, and steady-state PL spectra in the upper insets.

To comprehend the variation in X-ray response, the first crucial material property to consider is the energy required to create an electron–hole pair (*W*) upon X-ray illumination. This can be determined by the bandgap energy (*E*_g_) using the equation *W* = 2*E*_g_ + 1.43.^48^ To determine *E*_g_, UV-Vis diffuse reflectance spectra of the phase-pure and dual-phase CsPbBr_3_ MCs are measured, after which the corresponding absorbance values are calculated using the Kubelka–Munk theory. Tauc plots allow us to quantify and study the change in the bandgap energy (Fig. 3). The results show that both the phase-pure and dual-phase crystals exhibit an absorption edge at 530 nm, corresponding to a direct bandgap of 2.3 eV, which agrees with the literature for phase-pure CsPbBr_3_ crystals.^49^ No clear difference between the phase-pure and dual-phase crystals can be seen at the absorption onset, as the contribution of the absorption edge and bandgap of the CsPb_2_Br_5_ phase is expected to be around 3.40 eV (365 nm) and is therefore hidden in the reflectance spectrum.^50^

Steady-state photoluminescence (PL) spectra are recorded to identify possible changes in the electronic states due to the introduction of the CsPb_2_Br_5_ phase. For the phase-pure CsPbBr_3_ perovskite crystals, the steady-state PL spectra show two PL maxima at 530 nm (2.3 eV) and 550 nm (2.25 eV) (PLQY < 1%), with the first peak being the most intense (Fig. 3(a)). Similar double-peak emission has already been observed in melt-grown CsPbBr_3_ single crystals,^28^ as well as in solution-grown single CsPbBr_3_ crystals.^51^ The first PL peak almost coincides with the bandgap value of 2.30 eV and can therefore be attributed to near-band-edge emission. However, the second PL peak exhibits a Stokes shift of 20 nm (0.09 eV). This can either be due to the existence of sub-bandgap states arising from defects or due to phonon-assisted processes.^52,53^ For the dual-phase crystals, the same peaks can be observed, however the peak at 550 nm is more intense compared to the one at 530 nm (PLQY < 1%) (Fig. 3(b)). This can be explained by Rashba splitting^54^ or by the introduction of the second phase, which creates new defects in the perovskite lattice and therefore enhances the intensity of the second PL peak attributed to the radiative recombination through defect states.^55–57^ To identify the exact cause of the PL peak splitting, further in-depth investigations are provided below.

**Fig. 4:**
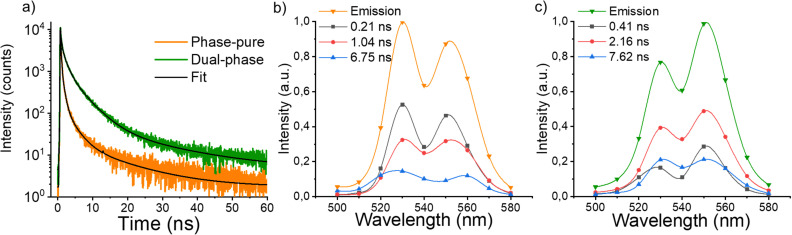
(a) Time-resolved luminescence decay traces and their corresponding fit (black) of phase-pure MCs (orange) and dual-phase MCs (green) (*λ*_exc_ = 460 nm, *λ*_em_ = 530 nm). Decay-associated spectra (DAS) resulting from TCSPC measurements of (b) phase-pure MCs and (c) dual-phase MCs (right).

The second crucial material property to consider is the charge carrier transport, as defined by the *μτ*-product, where *μ* is the carrier mobility and *τ* the fundamental carrier lifetime. To gain insight into the carrier lifetime, we performed time-resolved PL measurements in the ps–ns time range using time-correlated single photon counting (TCSPC) at different emission wavelengths. The PL decays of the phase-pure and dual-phase MCs (Fig. 4(a)) can be fitted with three main components (Table S1, ESI†). However, the complexity of the system does not allow us to indisputably assign each component to a specific process. Therefore, we proceed with a calculated average weighted lifetime 〈*τ*〉, resulting in 1.44 ns for the phase-pure MCs and 3.28 ns for the dual-phase MCs, which is more than two times longer. For both samples, the decay associated spectra (DAS) (Fig. 4(b) and (c)) show that their components contribute to the steady-state emission with different weights. In the phase-pure sample, the most dominant contribution arises from the 0.21 ns component, while the 2.16 ns component is more dominant for the dual-phase, resulting in the higher average weighted lifetime. This might be explained by the introduction of the 2D CsPb_2_Br_5_ structure, which is correlated with more defect states, as was already proposed in steady-state PL by the enhanced intensity of the second peak. Perovskite materials are generally defect tolerant, resulting in the formation of shallow (near the band edge) defect levels. These defects slow down the electron–hole recombination kinetics due to trapping and de-trapping, leading to longer lifetimes.^58–61^ Furthermore, the DAS of the dual-phase MCs (Fig. 4(c)) indicates that, over time, the relative contribution of the second peak associated with defects is decreasing, confirming the de-trapping process. We thus believe that, to some extent, the formation of shallow defect levels in the perovskite lattice structure can be advantageous in applications for energy conversion, in particular from the perspective of charge carrier lifetime.

**Fig. 5:**
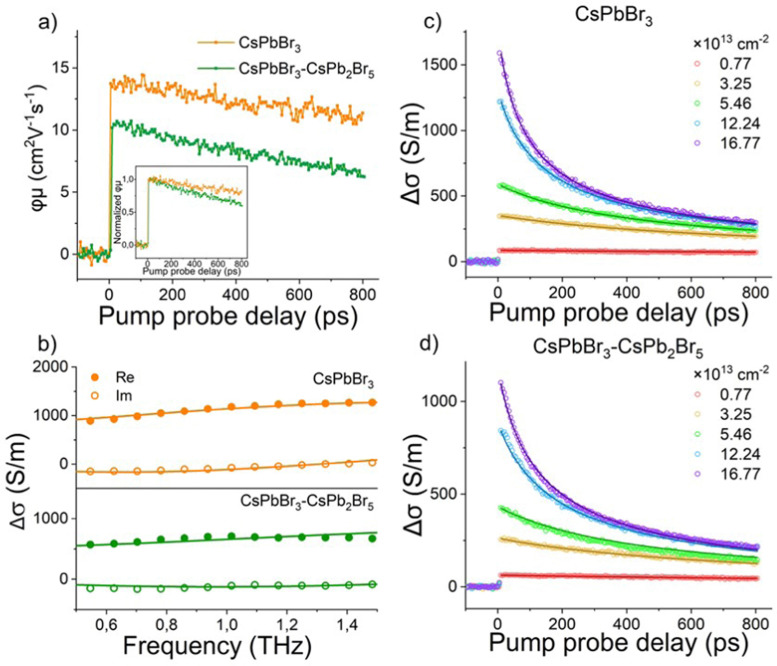
(a) Comparison of photoconductivity dynamics at an incident pump photon density of 0.77 × 10^13^ cm^−2^. Inset shows the normalized dynamics. (b) Frequency-resolved photoconductivity spectra for the phase-pure and dual-phase MCs, measured at pump–probe delay time of 5 ps and at the incident pump photon density of 1 × 10^14^ cm^−2^. Fluence-dependent OPTP dynamics of (c) phase-pure and (d) dual-phase MCs. All the measurements are conducted using an excitation wavelength of 400 nm at room temperature in dry nitrogen atmosphere.

To study the influence of the secondary phase on the electrical transport of CsPbBr_3_ thin films, we conducted ultrafast and contact-free THz time-domain spectroscopy (THz-TDS) with sub-ps time resolution. In the optical pump-THz probe (OPTP) experiment, the perovskite thin film (with a typical thickness of ∼200 nm) was first photoexcited by an optical pulse (400 nm, ∼100 fs pulse duration) to promote electrons from the valence band to the conduction band. Subsequently, a single-cycle THz pulse with electric field *E* transmits through the sample to probe the pump-induced change in the conductivity, *i.e.*, photoconductivity Δ*σ*. Δ*σ* is proportional to the relative attenuation of the THz electric field −Δ*E*/*E*_0_ (Δ*E* = *E*_pump_ − *E*_0_, with *E*_pump_ and *E*_0_ being the transmitted THz electric field with and without photoexcitation, respectively).^62^ By varying the pump–probe delay time *t*_p_, the photoconductivity dynamics can be obtained. In principle, Δ*σ* is proportional to the product of photo-generated charge carrier density (*N*, *N = N*_abs_*φ*, with *N*_abs_ = absorbed photon density and *φ* = the photon-to-free carrier quantum yield) and carrier mobility (*μ*), following: Δ*σ* = *Neμ* = (*N*_abs_*φ*)*eμ*. In Fig. 5(a), we plot and compare the product of charge carrier mobility and photon-to-free carrier quantum yield *φμ* (or equivalently Δ*σ*/(*N*_abse_)) for both the CsPbBr_3_ and CsPbBr_3_–CsPb_2_Br_5_ thin films. The dual-phase MCs show ∼24% decrease in the *φμ* product when compared to that of phase-pure CsPbBr_3_ samples. To further investigate which change (*e.g.*, mobility or free carrier density) dominates the reduced *μφ*, we measured the frequency-resolved photoconductivity spectra by Fourier-transforming the time-domain THz field Δ*E*(*t*) and *E*_0_(*t*), at the same incident pump fluence. The obtained spectra in Fig. 5(b) can be adequately described by the Drude–Smith model:^63^
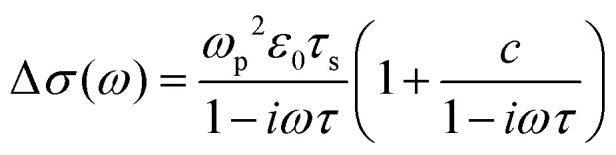


This model describes the free carrier transport with a preferential backscattering from *e.g.* the grain boundary. Here, parameter *c* (−1 < *c* < 0) is introduced to account for the extent of backscattering. For *c* = −1, the charge carriers experience 100% backscattering, while for *c* = 0, the Drude–Smith model is simplified to the Drude model. *ω*_p_ is the plasma frequency which is directly related to the carrier density *N* through 
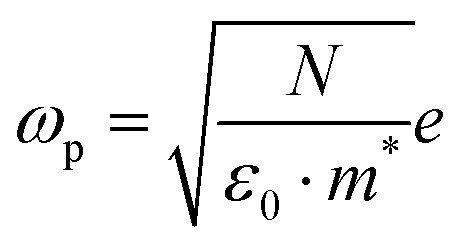
 (m* is the effective mass), *ε*_0_ the vacuum permittivity, *τ*_s_ the scattering time and ω the angular frequency. From the fitting, we extract nearly the same plasma frequency and parameter *c* for both samples (62.1 ± 1.1 THz and −0.68 ± 0.01 for CsPbBr_3_, 60.7 ± 3.6 THz and −0.69 ± 0.01 for CsPbBr_3_–CsPb_2_Br_5_), while the scattering time of CsPbBr_3_–CsPb_2_Br_5_ (51 ± 5 fs) is ∼30% lower than that of CsPbBr_3_ (73 ± 2 fs). When considering the mobility at the dc limit (
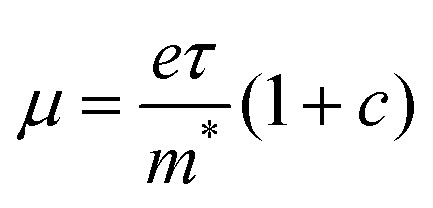
), the reduction of charge mobility in CsPbBr_3_–CsPb_2_Br_5_ mainly originates from the enhanced scattering rate (= 1/scattering time).

To gain further insights on the charge transport properties, we conducted fluence-dependent OPTP dynamics for both samples, and globally fitted them by the coupled rate equation for recombinations (Fig. 5(c) and (d)):^64^
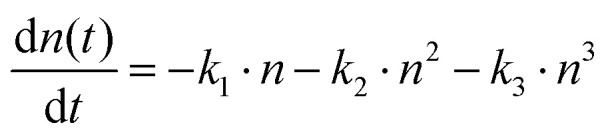
in which *n*(*t*) is the time-dependent carrier density. Here we assume the decay in the photoconductivity stems from the recombination, *e.g.* decay in the charge carrier population *n*, which should be valid for beyond-ps dynamics where charge carriers are expected to relax to the band edge. *k*_1_ is the monomolecular recombination rate constant which arises from charge trapping; *k*_2_ represents the bimolecular recombination rate constant which represents the free electron–hole pair recombination, and *k*_3_ stands for the Auger recombination rate constant. The first-order recombination is expected to strongly depend on the phase composition, and to increase for less pure phases, due to the likely reduced domain sizes and introduced defect states. The higher-order recombinations, on the other hand, mostly depend on local charge carrier densities, and are therefore much less susceptible to the details of the sample composition. Indeed, as shown in Table 1, *k*_2_ and *k*_3_ are comparable for both samples, however, a significant difference in *k*_1_ is observed, suggesting a higher density of defect states in CsPbBr_3_–CsPb_2_Br_5_. In line with our speculation and both static and time-resolved PL, introducing a lower dimensional side phase (CsPb_2_Br_5_) seems to induce additional shallow defect states, contributing to a prolonged lifetime, while to some extent reducing the charge mobility. This can be advantageous in high-voltage X-ray detection as it prevents excess charges from accumulating at the selective contacts.^65^ Furthermore, the dual-phase MCs exhibit a low dark current in X-ray detection measurements, suggesting a suppressed ion migration by increasing the ion migration energy barrier.^47^ All these aspects avoid the building up of unwanted polarization in the device configuration,^42^ resulting in an improved local charge extraction.

**Table 1:** Recombination rate constants of phase-pure and dual-phase MCs

Sample	*k*_1_ (s^−1^)	*k*_2_ (cm^3^ s^−1^)	*k*_3_ (cm^6^ s^−1^)
CsPbBr_3_	4.09 × 10^7^	3.70 × 10^−10^	6.75 × 10^−28^
CsPbBr_3_–CsPb_2_Br_5_	2.30 × 10^8^	4.57 × 10^−10^	6.75 × 10^−28^

## Conclusion

In this work, we have demonstrated a scalable deposition method for creating uniform, crack-free, and flexible films consisting of perovskite MCs. Furthermore, we investigated how the implementation of the 2D CsPb_2_Br_5_ phase into CsPbBr_3_ microcrystals affects their structural and optical properties. Rietveld refinement confirms the presence of ∼20% of Pb-vacancies in the dual phase, which eventually increases the ion migration energy barrier, thereby promoting its stability. Corresponding proof-of-concept devices were tested for sensitivity upon X-ray illumination, and it was found that the dual-phase sample demonstrated improved X-ray detection at higher voltage regimes. This enhanced stability provides a promising avenue for future improvements in fast charge extraction and X-ray image resolution. Time-resolved photoluminescence measurements revealed a prolonged lifetime of the dual-phase sample due to the presence of shallow defects that slow down electron–hole recombination kinetics. THz measurements confirmed the presence of additional defects, as indicated by the partially reduced carrier mobility. This is beneficial at higher voltages as it prevents the accumulation of an excess of charges at the contacts. Moreover, the dual-phase CsPbBr_3_–CsPb_2_Br_5_ MCs exhibit a much lower dark current, linked to suppressed ion migration, which, in combination with the formed Pb-based defect states, prevents the building up of polarization in the device configuration. These findings suggest that the introduction of Pb-defects may not be as detrimental as expected and could potentially serve as an effective means to optimize X-ray detection, as well as other high-voltage demanding energy conversion applications.

## Author contributions

L. C. conducted most of the experiments and their analysis, assisted by R. V. B., and wrote the manuscript under the supervision of B. P. and E. D. J. V. completed the time-resolved spectroscopy. R. A. S. assisted in XRD Rietveld refinement and calculations of the lattice parameters. G. R. and L. S. analyzed the time-resolved spectroscopy data. H. Z. performed THz measurements and analysis under the supervision of H. I. W. and M. B. L. C. and D. V. performed X-ray sensitivity measurements. The research line presented in the manuscript was conceived by E. D.

## Conflicts of interest

The authors declare no competing financial interests.

## Supplementary Material

TC-012-D3TC01903A-s001
