# Supplementary material for: Phase-engineering compact and flexible CsPbBr3 microcrystal films for robust X-ray detection
Source: J Mater Chem C Mater. 2023 Dec 11;12(2):655–63. doi: 10.1039/d3tc01903a (PMC10766070; doi:10.1039/d3tc01903a)
Supplement: TC-012-D3TC01903A-s001 [file TC-012-D3TC01903A-s001.pdf]

Electronic Supplementary Material (ESI) for Journal of Materials Chemistry C.
This journal is © The Royal Society of Chemistry 2023

Supporting Information

Phase-Engineering Compact and Flexible $CsPbBr_3$ Microcrystal Films for Robust X-ray Detection

Lotte Clinckemalie[1], Bapi Pradhan[1], Roel Vanden Brande[1], Heng Zhang[2], Jonathan Vandenwijngaerden[1], Rafikul Ali Saha[3], Giacomo Romolini[1], Li Sun[1], Dirk Vandenbroucke[4], Mischa Bonn[2], Hai I. Wang[2], Elke Debroye[1]**

[1] Department of Chemistry, KU Leuven, Celestijnenlaan 200F, 3001 Leuven, Belgium

[2] Max Planck Institute for Polymer Research, 55128 Mainz, Germany

[3] cMACS, Department of Microbial and Molecular Systems, KU Leuven, Celestijnenlaan 200F, 3001 Leuven, Belgium

[4] Agfa-Gevaert, Septestraat 27, B-2640 Mortsel, Belgium

** Corresponding authors*

Detailed description of experimental procedures

Material synthesis:

All precursor chemicals were obtained commercially from either Alfa Aesar (CsBr, 99%), Thermo Scientific (HBr, pure, 48 w%) or Sigma-Aldrich ($PbBr_2$, 98%) and used without any further purification. The $CsPbBr_3$-$CsPb_2Br_5$ microcrystals are synthesized by first separately dissolving a stoichiometric molar ratio of CsBr (5 mmol) and $PbBr_2$ (5 mmol) in respectively 1 mL of demi H_2O and 4 mL of 48 w% HBr by sonication. The Cs-H_20 solution is then dropwise added over 10 minutes to the Pb-HBr solution under ambient conditions and constant stirring. Finally, the crystals are centrifuged (5 min, 3500 rpm), washed with ethanol and vacuum dried at 60 °C (Figure S1).

Thin films for THz measurements were prepared by spin-coating a 0.5 M DMSO solution of both the dual-phase and phase-pure MCs. The solutions were prepared by dissolving 0.290 g of the MCs in 1 mL of dry DMSO at around 70 °C. The solution was then filtered with a 0.20 µL pore PTFE hydrophilic filter. For obtaining the thin film, 70 µL of the perovskite solution was spin-coated on the fused silica glass, in a glovebox. Subsequent spin-coating at 1000 rpm for 10 s, followed by 60 s at 3000 rpm, with the addition of 125 µL of chlorobenzene 35 s before the end of the program, resulted in the desired thin film after solvent evaporation (for 10 min on a hot plate at 60 °C). Both of the films were annealed outside the glovebox for 30 min at 150 °C. The films were stored in the dark under an inert atmosphere until further measurements.

Material characterization:

Scanning electron microscopy (SEM) was used to obtain images of the crystals. They were recorded on a FEI-Q FEG250 instrument at an acceleration voltage of 10 and 15 kV.

X-ray diffraction (XRD) patterns were obtained on a Malvern PANalytical Empyrean diffractometer, equipped with a PIXcel3D solid-state detector using a Cu anode (Cu $K_{\alpha1}$: 1.5406 Å; Cu $K_{\alpha2}$: 1.5444 Å). The powder samples were loaded onto a 96-well sample holder and X-ray diffractograms were recorded at room temperature within a 1.3 - 45° 2θ range using a step size of 0.013°. The X-ray-diffraction (XRD) data were analyzed by using the Rietveld technique and refinements were done using the FULLPROF program.[1] We refine lattice parameters and crystallographic positions for all elements using the "Thompson-Cox-Hastings pseudo-Voigt * Axial divergence asymmetry" peak shape, ensuring an exquisite match between experimental data and the refined peak shape. In order to achieve a precise alignment between the observed XRD peaks and the calculated data, we carefully adjust the full width at half maximum (FWHM) by refining the U, V, W parameters, along with the shape parameters. For dual phase sample, during XRD analysis, we consider two phases and determine their respective fractions through Rietveld refinement. Radiation damage of phase pure and mixed phase films has been tested at the ESRF ID09 beamline (X-ray energy 36.75 keV/mm^2, spot size 40 µm×10 mm).[2]

X-ray detection measurements were performed by exposing the devices for 30 seconds to a Pantak-Seifert X-ray source with tungsten anode (maximum X-ray photon energy 70 keV, peak intensity 30 keV) in the configuration of PEN/ITO/perovskite/conductive silicone. The X-ray spot size was about 3 mm and the X-ray dose rate was carefully calibrated with a RaySafe Model Solo R/F dosimeter. A Keithley 2000 was used to apply the bias voltage and to record both the dark- and response current. All the X-ray response characterization was conducted in the dark.

UV-Vis Diffuse Reflectance data were recorded between 400 and 800 nm with a step of 1 nm via a PerkinElmer Lambda 950 UV-vis spectrophotometer equipped with an integrating sphere at ambient temperature. $BaSO_4$ powder was used as reference. The diffused reflectance (R) was converted to F(R) using the Kubelka–Munk function: $F(R) = (1 - R)^2/2R$.

Steady-state photoluminescence (PL) excitation-emission maps were measured with an excitation range between 250 nm and 600 nm with a step of 5 nm and an emission range between 300 and 800 nm with a step of 5 nm via an Edinburgh FLS980 fluorimeter.

Time-correlated single photon counting (TCSPC) was used to determine the fluorescence decay times at the nanosecond time scale. The frequency-tripled output (266 nm, 8.18 MHz, 2 ps FWHM) of a mode-locked Ti:Sapphire laser (Tsunami, Spectra Physics) was used as excitation source. The linearly polarized excitation light was rotated to a vertical direction using a Berek compensator (New Focus) in combination with a polarization filter and directed onto the samples. The samples in powder form were placed in a quartz cuvette (1 mm path length), sealed by a Teflon stopper, and then mounted on the device. The emission was collected under 90° with respect to the incident light and guided through a polarization filter that was set at the magic angle (54.7°) with respect to the polarization of the excitation beam. The fluorescence was spectrally resolved by a monochromator (Sciencetech 9030, 100 nm focal length, wave- length accuracy 0.3 nm), and detected by a microchannel plate photomultiplier tube (MCP-PMT, R3809U-51, Hamamatsu). A time-correlated single photon timing PC module (SPC 830, Becker & Hickl) was used to obtain the fluorescence decay histogram in 4096 channels. The decays were recorded with 10000 counts in the peak channel, in time windows of 12 ns corresponding to 2.92 ps per channel and analyzed individually with a time-resolved fluorescence analysis (TRFA) software based on iterative reconvolution of the data with the instrumental response function (IRF). The full width at half- maximum (FWHM) of the IRF was typically in the order of 42 ps.

Terahertz photoconductivity measurements provide a contact-free means for photoconductivity measurements. Using an ultrashort visible laser pulse (400 nm, FWHM ≈ 50 fs), charge carriers were

optically injected into the conduction bands. Subsequently, the conductivity of the photogenerated charge carrier was probed by freely propagating single-cycle THz electromagnetic transients with 1–2 ps duration. The quickly oscillating terahertz field E(t) accelerated the charges, which, in turn, modifies the terahertz pulse. The relative modification $-\Delta E/E(t)$ of the THz field can be directly measured and related to the photoconductivity σ. The analysis was typically performed in the frequency domain by Fourier transformation, that is, $\sigma(\omega) \approx -\Delta E/E(\omega)$. The photoconductivity as a function of time delay t_d between excitation and THz pulse $\sigma(\omega;t_d)$ was a complex value, and its real part, $Re[\sigma(\omega;t_d)]$, corresponding to the changes in the peak intensity of THz pulse following photoexcitation, was directly related to the macroscopic transport parameters in electrical measurements, by $Re[\sigma(\omega\ ;\ t_d)] = N_{pump} \times e \times \mu(\omega\ ;\ t_d)$ where N_{pump} and $\mu(\omega;\ t_d)$ were the time-dependent carrier density and mobility following photoexcitation, respectively. Terahertz spectroscopy thus provides information on charge carrier dynamics on a timescale complementary to that obtained using time-resolved PL measurements (sub-ps versus ns).

Results and discussion

a) b)

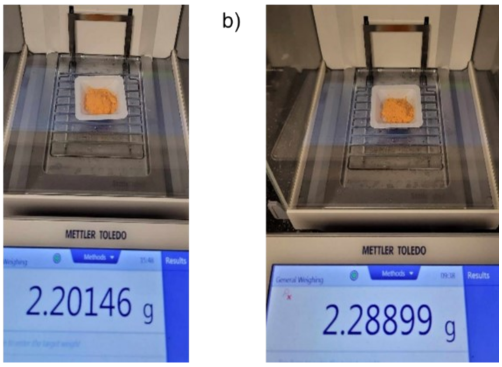

Figure S1. Gram scale reaction products of as synthesized a) phase-pure $CsPbBr_3$ and b) dual-phase $CsPbBr_3$-$CsPb_2Br_5$ powder microcrystals.

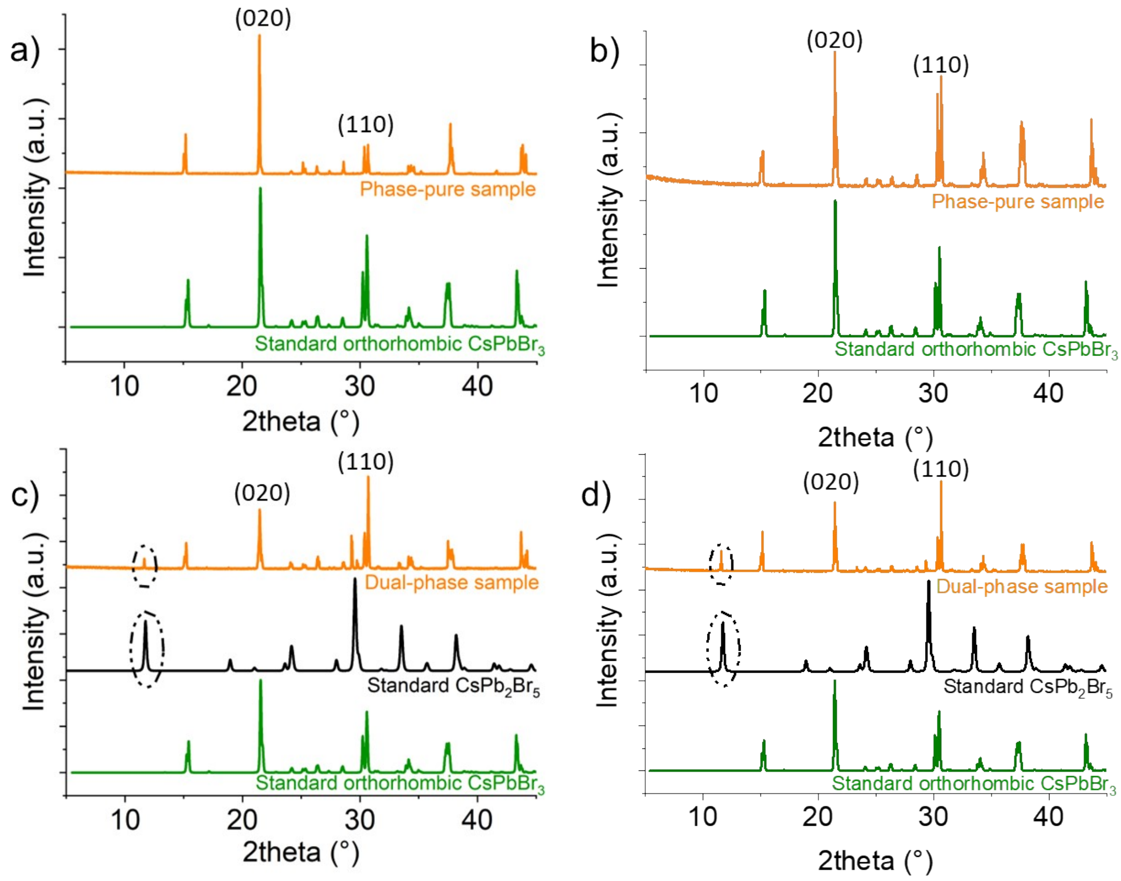

Figure S2. XRD pattern revealing the crystal structure of the phase-pure orthorhombic $CsPbBr_3$ and dual-phase $CsPbBr_3$-$CsPb_2Br_5$ of the powder sample (a, c) and the thick-film (b, d) respectively.

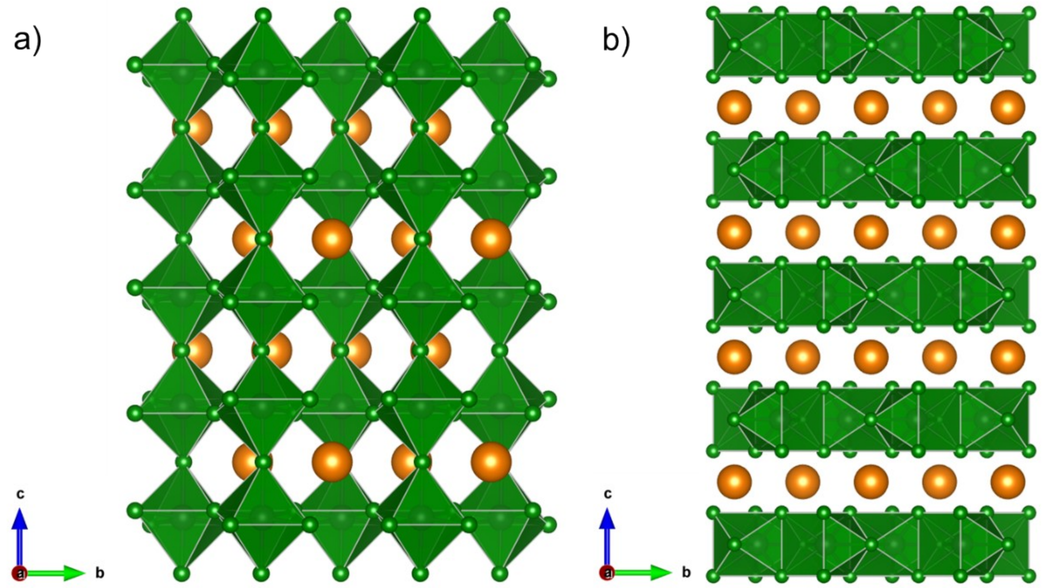

Figure S3. Illustration of the crystal structures of a) orthorhombic $CsPbBr_3$ and b) (2D) tetragonal $CsPb_2Br_5$.

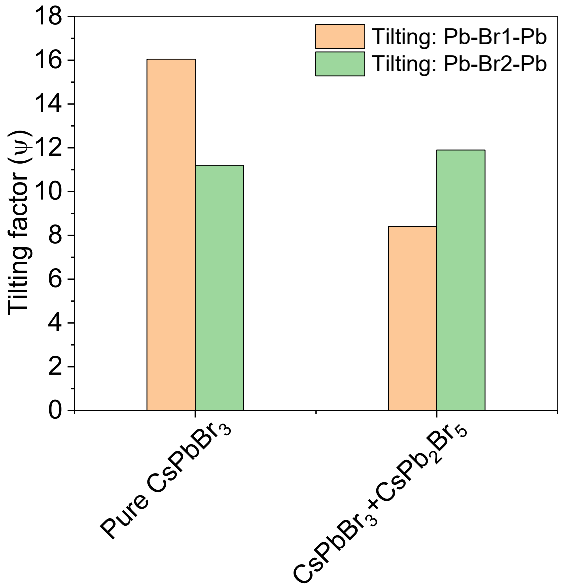

Figure S4. Octahedral tilting factor of $CsPbBr_3$ phase for the two different samples, pure $CsPbBr_3$ and dual phase powders. Orange bar is corresponding to Pb-Br1-Pb bond angle and the green bar is corresponding to Pb-Br2-Pb bond angle.

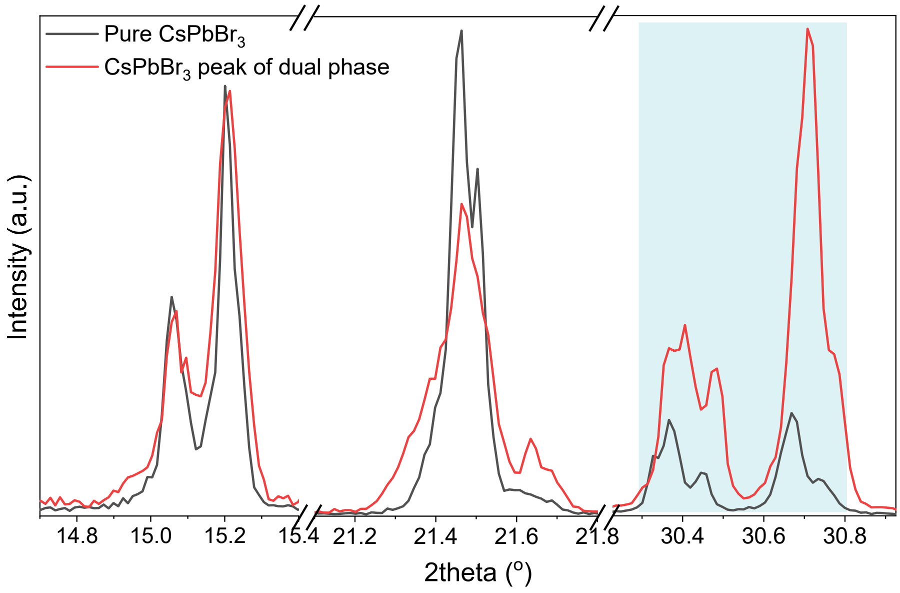

Figure S5. Comparison of XRD peak position shifts of the pure $CsPbBr_3$ phase with dual phase powder samples.

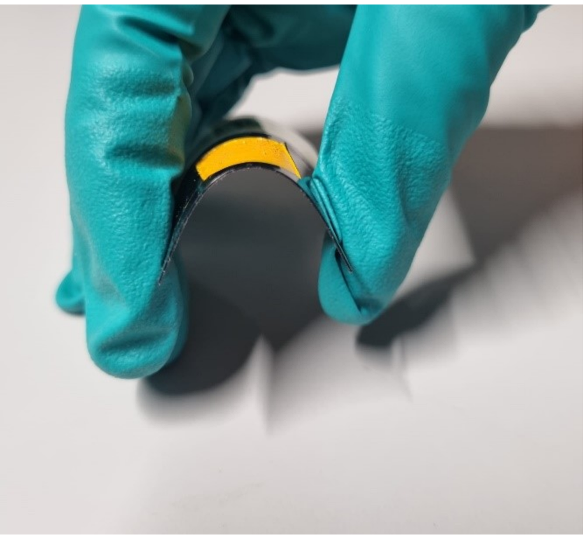

Figure S6. Photograph of a compact thick film fabricated in the lab, showing its flexibility.

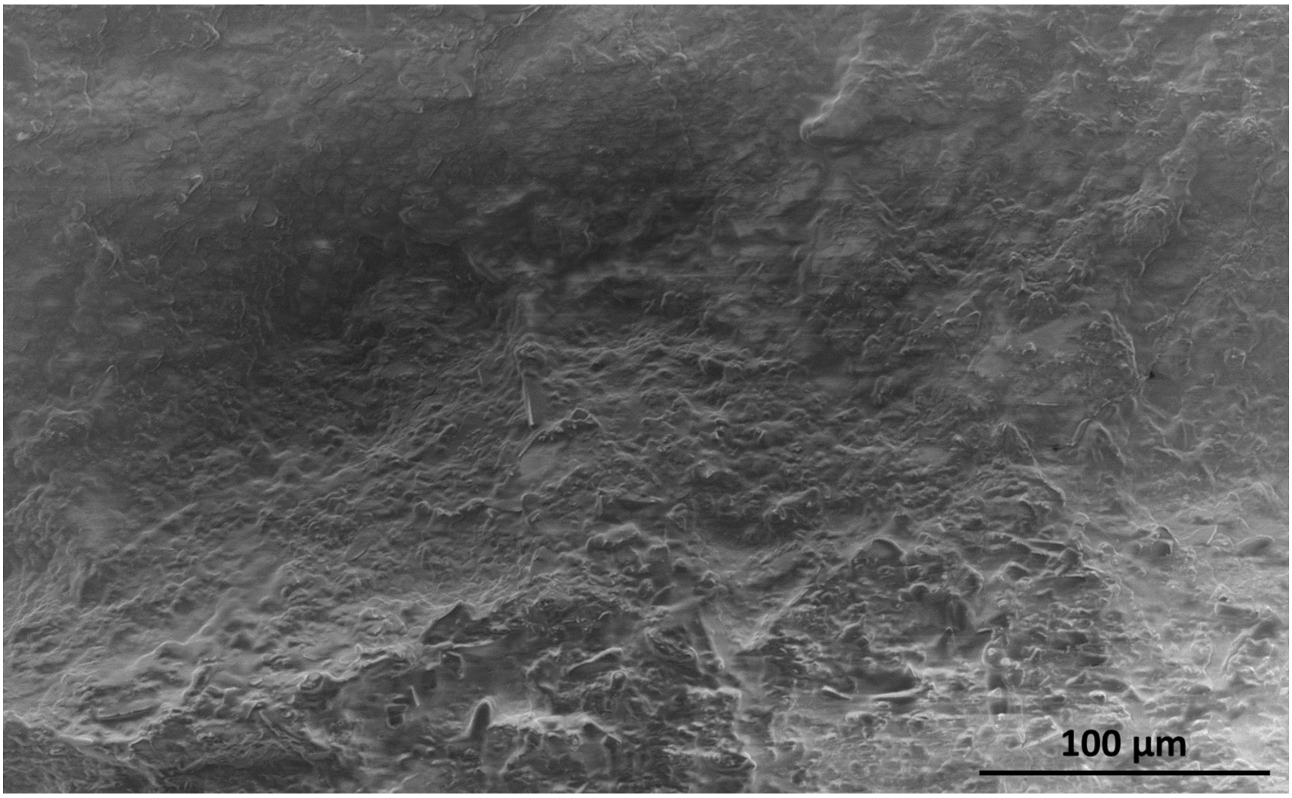

Figure S7. Scanning electron microscopy (SEM) image of the compact $CsPbBr_3$ film.

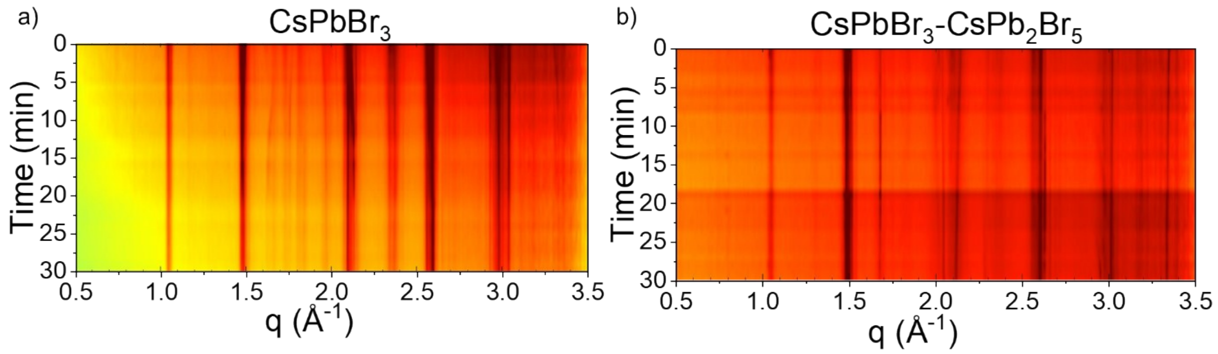

Figure S8. X-ray beam damage test: 2D waterfall plot of synchrotron-based GIWAXS of a) the phase-pure orthorhombic $CsPbBr_3$ and b) dual-phase $CsPbBr_3$-$CsPb_2Br_5$ films. The thin films were exposed to a 14.7 keV X-ray beam for 30 min with continuous GIWAXS measurements at every 1 min intervals. In the dual-phase $CsPbBr_3$-$CsPb_2Br_5$ film, the primary phase remains consistent throughout the entire 30 min X-ray exposure. However, in the phase-pure orthorhombic $CsPbBr_3$ film, a broad peak around 2.1 $Å^{-1}$ splits into two peaks presumably due to beam damage, while the main phase remains unaffected.

Table S1. Time components of the luminescence decay (λ_{exc} = 460 nm, λ_{em} = 530 nm) of the phase-pure and dual-phase MCs as fitted by a global analysis. It should be noted that the value of τ_1 (0.030 ns) is lower than the resolution of the instrument (0.042 ns) and is thus disregarded during further examination of the decays.

**Time components**	**Phase-pure MCs**	**Dual-phase MCs**
τ_1	0.030 ns	0.030 ns
τ_2	0.21 ns (**52.8%** contribution)	0.41 ns (21.4% contribution)
τ_3	1.04 ns (32.5% contribution)	2.16 ns (**51.2%** contribution)
τ_4	6.75 ns (14.7% contribution)	7.62 ns (27.4% contribution)

References:

1. Carvajal, J. R. Physica B 192, 55 (1993).

2. Pradhan, B., Saha, R. A., & Zhang, R. (2026). Visualization of dynamic polaronic strain fields in shape-tuned nanocrystalline CsPbBr3 perovskites [dataset]. European Synchrotron Radiation Facility. doi.org/10.15151/ESRF-ES-1345034448.
